# The role of flower pollen extract in managing patients affected by chronic prostatitis/chronic pelvic pain syndrome: a comprehensive analysis of all published clinical trials

**DOI:** 10.1186/s12894-017-0223-5

**Published:** 2017-04-21

**Authors:** Tommaso Cai, Paolo Verze, Roberto La Rocca, Umberto Anceschi, Cosimo De Nunzio, Vincenzo Mirone

**Affiliations:** 1 Department of Urology, Santa Chiara Regional Hospital, Trento, Italy; 2 Department of Urology, University of Naples, Federico II, Naples, Italy; 3 Department of Urology, Ospedale Sant’Andrea, Sapienza University of Rome, Rome, Italy

**Keywords:** Chronic pelvic pain syndrome, Inflammatory chronic pelvic pain syndrome, Prostatitis syndrome, Chronic prostatitis symptom index, Pollen extract

## Abstract

**Background:**

Chronic prostatitis/chronic pelvic pain syndrome (CP/CPPS) is still a challenge to manage for all physicians. We feel that a summary of the current literature and a systematic review to evaluate the therapeutic efficacy of flower pollen extract would be helpful for physicians who are considering a phytotherapeutic approach to treating patients with CP/CPPS.

**Methods:**

A comprehensive search of the PubMed and Embase databases up to June 2016 was performed. This comprehensive analysis included both pre-clinical and clinical trials on the role of flower pollen extract in CP/CPPS patients. Moreover, a meta-analysis of available randomized controlled trials (RCTs) was performed. The NIH Chronic Prostatitis Symptom Index (NIH-CPSI) and Quality of Life related questionnaires (QoL) were the most commonly used tools to evaluate the therapeutic efficacy of pollen extract.

**Results:**

Pre-clinical studies demonstrated the anti-inflammatory and anti-proliferative role of pollen extract. 6 clinical, non-controlled studies including 206 patients, and 4 RCTs including 384 patients were conducted. The mean response rate in non-controlled studies was 83.6% (62.2%-96.0%). The meta-analysis revealed that flower pollen extract could significantly improve patients’ quality of life [OR 0.52 (0.34-.0.81); *p* = 0.02]. No significant adverse events were reported.

**Conclusion:**

Most of these studies presented encouraging results in terms of variations in NIH-CPSI and QoL scores. These studies suggest that the use of flower pollen extract for the management of CP/CPPS patients is beneficial. Future publications of robust evidence from additional RCTs and longer-term follow-up would provide more support encouraging the use of flower pollen extracts for CP/CPPS patients.

## Background

Chronic prostatitis has been described as one of the most common illnesses in men aged <50 year [1] with differing clinical presentations [2]. According to the classification of the National Institute of Health (NIH) [3], class III chronic prostatitis/chronic pelvic pain syndrome (CP/CPPS) is the most frequent category [4]. Symptoms such as pelvic pain, painful voiding and ejaculation and disturbed sexual functioning are common, often resulting in a significant impact on quality of life [5]. Recently, it has been established that the annual cost of a patient affected by prostatitis exceeds that of a patient with type 1 diabetes and that his quality of life is analogous to a patient with a heart attack or acute Crohn's disease [5]. Available therapies for CP/CPPS are not highly effective and require further in-depth analysis and consideration of such alternate strategies [6]. The traditional treatment of CP/CPPS is known as the “three A’s”: antibiotics, anti-inflammatory medications, and alpha blockers. The use of antibiotics remains controversial, especially due to the fact that bacteria cannot be isolated from the urogenital samples of CP/CPPS patients [7]. On the other hand, even if anti-inflammatory medications, aspirin or other NSAIDs such as ibuprofen can decrease pain, they can only be taken for a limited period of time due to their high prevalence of drug-related adverse effects. In other words, the standard treatment for CP/CPPS has not yet been definitively established [7]. In this scenario, even if phytotherapeutics seems to be an interesting option because of their generally low side effects, demonstrated efficacy, and high treatment compliance by patients, few compounds have been subject to scientific scrutiny and prospective controlled clinical trials [8, 9].

Over the last few years, interest in the use of flower pollen extract in the management of CP/CPPS has increased. Several clinical experiments show that flower pollen extract preparations may allow for a durable and marked symptom reduction in young men with CP/CPPS with improvement in semen quality and a significant reduction in the National Institutes of Health-Chronic Prostatitis Symptom Index (NIH-CPSI) score [10–13]. The most common pollen extracts used in clinical trials is Graminex® (Graminex® LLC, 95 Midland Road, Saginaw, MI 48638) that is a mixture of standardized extracts of rye grass pollen (Secale cereal), corn pollen (Zea mays), and timothy pollen (Phleum pretense). However, up to the present no comprehensive analysis of the current literature has been made so as to evaluate the tolerability and clinical efficacy of flower pollen extract in the management of patients affected by CP/CPPS.

### Aim of the present review

Herein we aim to analyse all published data on flower pollen extract’s role in the management of patients affected by CP/CPPS both in a pre-clinical and clinical setting, with particular attention given to the randomized clinical trials. Moreover, we aim to analyse all published studies in order to identify all clinical, laboratory and instrumental characteristics that are able to predict patients’ clinical response to the treatment.

### Research questions

We put forth two research queries:

1. Is flower pollen extract able to obtain significant pre-clinical data in order to justify its clinical use in the management of patients affected by CP/CPPS?
2. Is flower pollen extract able to improve overall and disease-specific quality of life of patients affected by CP/CPPS?

## Methods

### Types of studies

We have included pre-clinical studies regarding the effects of flower pollen extracts as a background and narrative review. Moreover, we have included clinical trials, randomized controlled trials, cohort, and case-control studies for our systematic review and meta-analysis. Editorials, commentaries, and review articles were used only for the background and the narrative review.

### Outcome measures

The primary outcome of the study was the improvement of disease-related quality of life in terms of clinical response to the treatment as defined by the investigators. Clinical response to the treatment was generally evaluated in terms of NIH-CPSI and SF-36 questionnaires. Moreover, the improvement of symptoms [urinary and sexual symptoms, in terms of the International Prostatic Symptoms Score (IPSS)] and other questionnaires were also considered as outcome measures, if used by the investigators.

### Risk of bias assessment

The risk of bias was performed by using the Newcastle-Ottawa Scale for risk of bias assessment [14].

### Search strategy and research methods

**Fig. 1:**
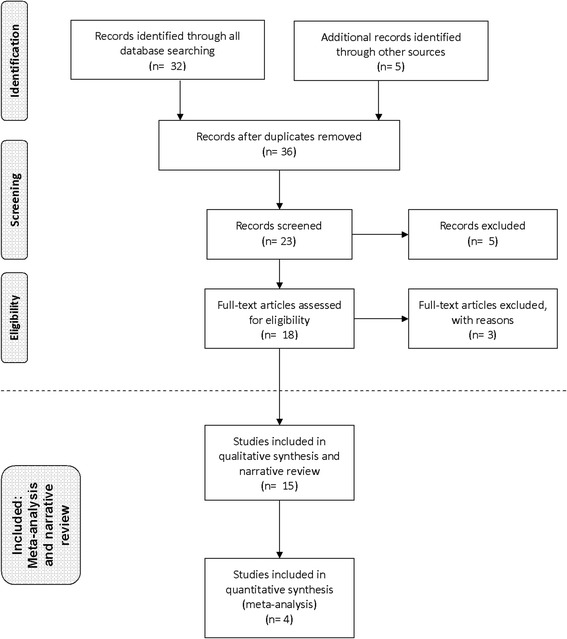
The figure shows the study flow chart in line with the PRISMA statement (http://www.prisma-statement.org/)

We performed a search of literature up to June 2016 using the Medline computerized database of the US National Library of Medicine. The Google Scholar database was used, too. The Medline search was carried-out using Medical Subject Headings and free text terms as follows: ‘pollen extract’, or “flower pollen extract” and ‘prostate’ (exploded) were combined with the terms: ‘treatment’ and ‘therapy’. Abstracts were not considered when full articles focusing on the same studies were available. Due to the limited number of pre-clinical studies published, we also included all non-English language papers as well. In cases of non-English language papers, the paper was included if the abstract was written in English and informative. Overlapping experiments have not been included because they were considered redundant. We considered as background information and as a comparative paper the latest review about the role of flower pollen extract in CP/CPPS patients by Wagenlehner FM published in 2011 [15]. From an initial literature search with pollen extract and prostatitis, a total of 23 extended papers were screened and 15 were selected and included in the present review. Finally, 10 clinical trials and 5 pre-clinical studies were analysed and are discussed in this review (Fig. 1). The Preferred Reporting Items for Systematic Reviews and Meta-Analyses (PRISMA) and Meta-analyses of Observational studies in Epidemiology (MOOSE) guidelines for the reporting of this present study was used in order to perform an accurate research check-list and report [16, 17]. The meta-analysis was performed using Review Manager 5.3 (Copenhagen: The Nordic Cochrane Centre, The Cochrane Collaboration, 2014) software. The inverse variance technique for the meta-analysis of the hazard ratios has been used. Due to the fact that the studies’ heterogeneity cannot be explained, a random-effects model has been employed which in fact involves an assumption that the effects being estimated in the different studies are not identical.

### Review methodology

Two authors performed the study selection independently (TC and PV). All disagreements were resolved by the senior author (VM). Titles and abstracts were used to screen for initial study inclusion. Full-text review was used where abstracts were insufficient to determine if the study met inclusion or exclusion criteria. Two authors (TC and PV) independently performed all data abstraction including evaluation of study characteristics, risk of bias, and outcome measures with independent verification performed by the senior author (VM). The limited number of the studies collected did not require any other authors.

## Results

### Pre-clinical evidence

Our literature search identified 5 pre-clinical studies (Table 1). Three “in vitro models” [18–20] and three animal models [12, 21, 22] were used. All the studies demonstrated/confirmed that pollen extracts show two important pharmacological effects: anti-inflammatory and anti-proliferative. Loschen et al. demonstrated that rye pollen is able to inhibit the synthesis of prostaglandin and leukotriene performing an anticongestive and anti-inflammatory effect on the prostate tissue [20]. Another aspect to take into account is the possible effect of pollen extract on other tissues that differ from those of prostate glands. In fact, Wagenlehner highlighted another pharmacological effect of pollen extract that can be considered as a therapeutic mechanism of this compound: its effect on smooth muscles [15]. In conformity with Wagenlehner [15], Nagashima demonstrated in an animal model that consecutive administration of flower pollen extracts increased significantly maximum pressure during micturition to promote micturition reflex [22].

**Table 1:** Summary of all pre-clinical studies

Author, year [reference]	Study type	Model	Compound used	Main study finding
Habib FK, 1990 [18]	In vitro study	Human prostate cancer cell line	pollen extract	- pollen extract is able to inhibit the prostate cancer cell growth (hormone-independent model)
Habib FK, 1995 [19]	In vitro study	Human prostate cancer cell line (DU145)	pollen extract	- pollen extract V-7 fraction is able to inhibit the prostate cancer cell growth
Kamijo T, 2001 [12]	Animal model	Rats	pollen extract	- pollen extract protects acinar epithelial cells and inhibits stromal proliferation in association with enhanced apoptosis
Loschen G, 1991 [20]	In vitro study	Microsomes (RBL-1 cells)	pollen extract	- pollen extract shows an anti-inflammatory and anti-proliferative therapeutic effect
Talpur N, 2003 [21]	Animal model	Rats	pollen extract *vs* serenoa repens	- pollen extract is able to influence prostatic hyperplasia via effects on androgen metabolism
Nagashima A, 1998 [22]	Animal model	Rats	pollen extract	- pollen extract increases the maximum pressure during urination to promote the urination reflex

### Anti-inflammatory effect

It was concluded that pollen extracts are able to inhibit prostaglandin and leukotriene synthesis and this effect is comparable to that of diclofenac and indomethacin and approximately 10 times higher than that of aspirin [20].

### Anti-proliferative effects

Several animal models showed that pollen extract has a possible effect on the prostate via the androgen metabolism [21]. Talpur and co-workers demonstrated that pollen extract decreased the size of the prostate in androgen-induced prostatic enlargement in rats [21]. The effect of pollen extract on prostate enlargement is due to the fact that a fraction of this compound is a powerful mitogenic inhibitor of fibroblastic and epithelial proliferation [15]. Moreover, Kamijo and co-workers found that pollen extract protects acinar epithelial cells and inhibits stromal proliferation in association with enhanced apoptosis [12]. Finally, several in vitro studies demonstrated that pollen extract is able to inhibit prostate cancer cell growth, as found by Habib [18]. This effect is even more pronounced in hormone-independent models, suggesting that there might be a place for pollen extract in the control of abnormal growth of hormone-insensitive cells [18].

### Clinical evidence and meta-analysis

We identified 10 clinical studies (Table 2) and selected 6 clinical non randomized trials [10, 11, 23–26] and 4 RCTs [13, 27–29]. All trials demonstrated that pollen extracts significantly improved total symptoms, pain, and QoL in patients with inflammatory CP/CPPS without severe side-effects. Cai et al. used Graminex® (Graminex® LLC, 95 Midland Road, Saginaw, MI 48638) in association with B vitamins for the treatment of inflammatory and non-inflammatory CP/CPPS [11, 27]. Wagenlehner used Cernilton for the treatment of inflammatory CP/CPPS [13], while Elist used Prostat/Poltit that contains 74 mg of highly defined extract of pollen from selected Graminae species [28]. Finally, Iwamura used an association of Cernitin T60 and Cernitin GBX [29].

**Table 2:** Summary of all clinical studies

Author, year [reference]	Study design	Patients number (response rate)	Controls Number (response rate)	Comparator	Outcomes measured
Buck AC. 1989 [23]	Prospective trial (phase II)	15 (86.6)	-	-	- pollen extract effective in the treatment of chronic prostatitis and prostatodynia.
Cai T. 2013 [11]	Prospective trial (phase II)	20 (90.0)	-	-	- pollen extract significantly improved total symptoms, pain, and QoL in patients with non-inflammatory CP/CPPS without severe side effects.
Cai T, 2014 [27]	Randomized controlled trial	41 (75.6)	46 (41.3)	ibuprofen	- pollen extract significantly improved quality of life of patients when compared with those treated with ibuprofen (treatment difference in the NIH-CPSI pain domain, -2.14 ± 0.51, P < 0.001; QoL scores, P = 0.002).
Elist J. 2006 [28]	Randomized controlled trial	30 (73.3)	28 (64.2)	Placebo	- pollen extract is superior to placebo in providing symptomatic relief in men with chronic nonbacterial prostatitis/chronic pelvic pain syndrome.
Iwamura H, 2015 [29]	Randomized placebo-controlled trial	50 (78.1)	50 (88.2)	Eviprostat (phytotherapeutic agent)	- pollen extract significantly reduced the symptoms of category III CP/CPPS without any adverse events, in terms of NIH-CPSI, IPSS, and QoL.
Jodai A, 1988 [24]	Prospective trial (phase II)	32 (75.0)	-	-	- pollen extract significantly reduced the symptoms in 75.0% of all treated patients.
Monden K. 2002 [25]	Prospective trial (phase II)	24 (91.6)	-	-	- pollen extract significantly reduced the symptoms of chronic prostatitis group
Rugendorff EW. 1993 [10]	Prospective trial (phase II)	90 (62.2)	-	-	- pollen extract significantly reduced the symptoms of category III CP/CPPS without any adverse events, in terms of urinary symptoms and QoL.
Suzuki T. 1992 [26]	Prospective trial (phase II)	25 (96.0)	-	-	- pollen extract significantly reduced the symptoms of prostatitis patients without any adverse events.
Wagenlehner FM. 2009 [13]	Randomized controlled trial	70 (70.6)	69 (49.3)	Placebo	- pollen extract significantly improved total symptoms, pain, and QoL in patients with inflammatory CP/CPPS without severe side-effects.

### Non-RCTs

As reported in Table 2, 6 clinical, non randomized trials including 206 patients were selected. The mean response rate in non-controlled studies was 83.6% (62.2%-96.0%). Cai and co-workers in a non-randomized clinical study reported a clinical response rate of 90%, demonstrating that pollen extract in association with vitamins significantly improved total symptoms, pain, and QoL in patients with non-inflammatory CP/CPPS without severe side effects [11]. The same results, in terms of clinical efficacy, were reported by Rugendorff [10] and Buck [23] in two non-randomized trials which reported a clinical response rate of 62.2% and 86.6%, respectively. Moreover, three studies by Japanese researchers demonstrated a high clinical response rate to pollen extract treatment in patients with both class IIIa and class IIIb CP/CPPS [24–26].

### RCTs and meta-analysis

**Fig. 2:**
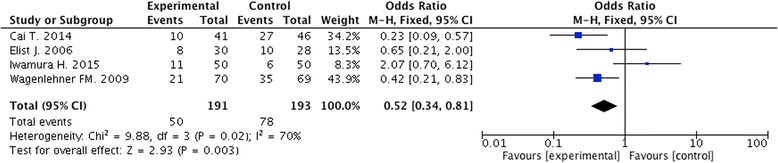
The figure shows forest plot of the effect of pollen extract on CP/CPPS patients in terms of clinical response rate

The mean response rate in RCTs was 74.4% (70.6%-78.1%). The latest RCT carried out by Iwamura and co-workers demonstrated a response rate of 78.1% in 50 patients affected by CP/CPPS after 8 weeks of treatment [29]. The authors defined the clinical response as a decrease in the NIH-CPSI total score by at least 25% [29]. They did not observe severe adverse events in any patients in their study [29]. On the other hand, Cai and co-workers, in a cohort of patients randomized to pollen extract or ibuprofen, reported a response rate of 75.6% in the flower pollen extract group [27]. Both class IIIa and class IIIb CP/CPPS patients were enrolled and, moreover, it was reported that adverse events were less frequent in the pollen extract group than in the ibuprofen group [27]. In the largest study, Wagenlehner and co-workers demonstrated a clinical response rate of 70.6% [13] in 139 patients affected by inflammatory CP/CPPS and treated for 12 weeks with flower pollen extract. They concluded that the beneficial effect continued to improve after 12 weeks’ treatment showing that pollen extract can be recommended for patients with inflammatory CP-CPPS for long-term treatment [13]. In 2006 Elist, by carrying out a double-blind study which included 60 patients with class IIIa or class IIIb CP/CPPS who were treated with flower pollen extract for 6 months, reported an overall clinical response of 73% [28]. All these 4 RCTs were used for the/included in our meta-analysis. We included 384 patients from 4 studies. The meta-analysis revealed that flower pollen extract could significantly improve patients’ quality of life [OR 0.52 (0.34-.0.81); *p* = 0.02]. Figure 2 shows the forest plot of the effect of pollen extract on CP/CPPS patients in terms of clinical response rate, as defined by the investigators.

### Sub-analysis on the basis of CP/CPPS type (class III a or b)

The analysis of the 4 RCT studies did not permit us to clearly identify which CP/CPPS sub-type was the best candidate to treat with the pollen extract. In this sense, the CP/CPPS class type is not able to predict patients’ clinical response to the treatment. Only one out of four studies enrolled inflammatory CP/CPPS (class A) [13], while the other three studies enrolled both class III a and b [27–29]. Cai and co-workers enrolled 25 patients with inflammatory CP/CPPS (type IIIa) and 62 type IIIb [27]. They found that in the pollen extract group patients affected by type IIIb CP/CPPS showed higher QoL results and a lower pain level following treatment in terms of the NIH-CPSI score (the NIH-CPSI score was 24.8 ± 1.8 at the enrolment versus 11.7 ± 1.7 at the follow-up visit; P < 0.001) when compared with type IIIa CP/CPPS patients [27]. Iwamura and co-workers enrolled 20 participents with class IIIa and 19 with class IIIb, without any reference to the difference between the two groups [29]. Finally, Elist did not report the results stratified by the CP/CPPS class [28]. In the two studies in which in which a data stratification according to class IIIa or b, 84 class A CP/CPPS and 30 class b had been treated with pollen extracts, while 80 class A CP/CPPS and 32 class b were considered as controls. The lack of data did not allow a significant analysis.

### Risk of bias assessment

The 4 RCTs included showed few risk of bias. Three studies contained both class IIIa and IIIb CP/CPPS patients, thus introducing the risk of a selection bias. Moreover, the RCT by Elist showed an important risk of a selection bias due to the fact that in this study patients between 20 and 60 years were included.

## Discussion

### Main findings

Pollen extract is a mixture of natural components, such as amino acids, carbohydrates, lipids, vitamins, phytosterols and minerals that have been introduced in urological practice for the treatment of CP/CPPS patients [15]. In this review and meta-analysis of 4 RCTs with low-to-moderate risk of bias, we found that the use of flower pollen extracts in the management of CP/CPPS patients is associated with a high rate of clinical response without any significant adverse events. Moreover, we found that in both class IIIa and class IIIb the use of pollen extract is able to obtain significant improvements in a patients’ QoL. These findings allow us to discuss several beneficial aspects of the role of pollen extract in the management of CP/CPPS patients. Firstly, upon consideration of the high clinical response rate of all included papers it was found that the mean response rate was high in both non-controlled [83.6% (62.2%-96.0%)] and in RCTs studies [74.4% (70.6%-78.1%)]. In analysing the reported encouraging results in terms of variations in NIH-CPSI and QoL scores, the following considerations should be taken into account:

- the proven anti-inflammatory, anti-proliferative effect of pollen extract
- the low rate of adverse events

All pre-clinical studies demonstrated that pollen extracts show an important anti-inflammatory effect due to the inhibition of prostaglandin and leukotriene synthesis [20]. Moreover, the dose-dependent, anti-inflammatory action of pollen extract in nonbacterial prostatitis in rats leading to decreased levels of interleukin-1b, interleukin-6 and a tumour necrosis factor, decreases glandular inflammation [15] has been demonstrated. The anti-inflammatory effect of pollen extract is approximately 10 times higher than that of aspirin [20] and did not lead to significant adverse events. This aspect is very important to highlight, due to the fact that the low prevalence of adverse effects correlates with a high patient compliance rate to the treatment. Moreover, several pre-clinical experiences demonstrated that flower pollen extract is able to inhibit 5a-reductase activity in the epithelium and stroma of the prostate in vitro, inhibiting the formation of dihydrotestosterone from testosterone [15]. This could be the reason for the improvements in urinary symptoms reported by the patients. However, the inhibition of 5a-reductase activity requires a long-term treatment as highlighted by several authors [15]. Even if a placebo effect was generally reported in patients treated with phytotherapeutic agents, in the 4 RCTs, clinically significant improvements were only observed in the pollen extract group and not in the placebo group. Finally, while Wagenlehner and co-workers found a decrease in leukocytes in post-prostate massage urine samples in both patients and controls [13], they did not find a significant difference between the two groups in terms of leukocyte number and for this reason leukocytes cannot be correlated with clinical success [13]. This aspect supports the hypothesis that the presence of inflammatory cells in the post-prostate massage urine sample is not a laboratory characteristic that is able to predict treatment response.

### Strengths and limitations of the present study

In this review we excluded all studies on the effect of pollen extract on patients affected by benign prostatic hyperplasia or other urological diseases that can determine symptoms. Moreover, we excluded all studies in which the dosage of the compound was indicated in the publication. For this reason, despite the latest review by Wagenlehner we have excluded the paper by Li [30]. On the other hand, the most important limitation of this review is the lack of a pharmacokinetic evaluation of pollen extract. As highlighted by Wagenlehner, pharmacokinetic studies on the absorption, distribution, metabolism or excretion of the active components of flower pollen extracts have not been performed [15]. This is due to the fact that it is not known which compounds are primarily responsible for clinical efficacy [15].

### Clinical implications

It is well known that there is no standard treatment or CP/CPPS to date. Amongst all the drugs and therapeutic approaches suggested and used, phytotherapeutic agents are those most widely prescribed in every day clinical practice with variable success. However, their use has only rarely been evaluated in suitable clinical trials. On the other hand, pollen extract has been sufficiently evaluated in preclinical and clinical studies [10, 13, 15]. Herein, we report encouraging results in terms of variations in NIH-CPSI and QoL scores in patients treated with pollen extracts indicating that the use of pollen extract appears to be safe and well tolerated by patients and, for this reason, the compliance to the treatment is high.

## Conclusion

In conclusion, based upon our study analysis, pollen extracts appear to be clinically beneficial as indicated by the significant improvements in terms of the NIH-CPSI and QoL scores of patients diagnosed with CP/CPPS. Moreover, this therapeutic approach has an excellent safety profile with limited reported adverse effects. Future publications containing robust evidence from additional RCTs and longer-term follow-up would provide doctors with more confidence regarding the use of flower pollen extracts for their CP/CPPS patients.

## Abbreviations

CP/CPPS: Chronic prostatitis/chronic pelvic pain syndrome
IPSS: International prostatic symptoms score
NIH: National institutes of health
NIH-CPSI: National institutes of health-chronic prostatitis symptom index
NSAIDs: Non-steroidal anti-inflammatory drugs
QoL: Quality of life
RCTs: Randomized clinical trials
SF-36: The short form (36) health survey
